# Smallpox Vaccination of Laboratory Workers at US Variola Testing Sites

**DOI:** 10.3201/eid2108.140956

**Published:** 2015-08

**Authors:** Sharon Medcalf, Laura Bilek, Teresa Hartman, Peter C. Iwen, Patricia Leuschen, Hannah Miller, Anne O’Keefe, Harlan Sayles, Philip W. Smith

**Affiliations:** University of Nebraska Medical Center, Omaha, Nebraska, USA

**Keywords:** smallpox vaccine, smallpox, vaccinia, laboratories, viruses, variola

## Abstract

To evaluate the need to revaccinate laboratory workers against smallpox, we assessed regular revaccination at the US Laboratory Response Network’s variola testing sites by examining barriers to revaccination and the potential for persistence of immunity. Our data do not provide evidence to suggest prolonging the recommended interval for revaccination.

The eradication of smallpox (variola) is arguably the greatest public health feat in the history of civilization. Smallpox was an infectious disease that plagued global health from the earliest documented settlements (1500 bce) through nearly the end of the 20th century (*1*). In 1980, the World Health Organization declared that smallpox was eradicated and paid homage to a large cadre of dedicated and tireless persons who collectively eliminated a disease that had killed one third of its victims for >3,500 years (*2*).

Despite concern about the existence of live smallpox virus housed in government research laboratories in the United States and the Soviet Union (later Russia), not until 2002 did the US government declare the resurgence of smallpox a credible biothreat (*1*). In the United States, the Department of Health and Human Services launched a national campaign to vaccinate volunteers from health care and public health professions. The 2003 National Smallpox Vaccination Program resulted in the vaccination of almost 40,000 volunteers in 9 months in the United States (*2*).

After the National Smallpox Vaccination Program ended in October 2003, experts at the Centers for Disease Control and Prevention (CDC; Atlanta, GA, USA) and members of the Advisory Committee on Immunization Practices (ACIP) met to determine future steps for response planning. These experts recommended that vaccinated persons from health care and public health professions be revaccinated only if a smallpox event occurred. However, laboratory workers handling orthopoxviruses at the proposed variola testing sites would need to be revaccinated every 3 years to maintain optimal immunity or protection. The ACIP admitted that this recommendation was based on “best available science, historic precedent, and expert opinion” (*3*). Although the ACIP recommendations do not address the frequency with which workers are exposed to orthopoxviruses, they imply that even periodic exposure to these viruses might warrant the protection that only vaccination can provide. Some orthopoxviruses remain extremely dangerous (high rates of illness and death) for humans (*4*).

During 2012, we further evaluated the need for revaccination of laboratory workers by examining barriers to revaccination and the potential for persistence of immunity in laboratory workers at the Laboratory Response Network (LRN) variola testing sites who had received at least 1 smallpox vaccination since 2003. Our intent was to balance the risks for rare exposures to the virus against the risks for severe adverse events from the vaccine.

## The Study

We used the LRN as a conduit to maintain the confidentiality and anonymity of the variola testing sites. A convenience sample of 45 laboratory workers completed an online survey developed by researchers at the University of Nebraska Medical Center (Omaha, NE, USA). Nonidentifying demographic information was collected, in addition to any adverse effects after vaccination and perceived barriers to revaccination. To determine a significant difference existed regarding the success (presence or absence of a “take” after vaccination) of the vaccine based on intervals between vaccines, we measured the mean interval (in years) between vaccinations. Finally, respondents were asked whether they worked with orthopoxviruses in their laboratories.

Respondents’ mean age was 46 years; they had worked a mean of 20.5 years in the laboratory setting. Eighty-four percent of respondents reported that the only adverse events from vaccination were related to the skin irritation caused by the occlusive dressings worn over the vaccination lesion. Sixty-seven percent listed a medical condition in themselves or a close household contact as the barrier to revaccination. The mean interval from first to second vaccination was 4.8 years for vaccinees who had a successful vaccine and 6.0 for those who did not. Statistical analysis demonstrated no significant difference (p = 0.149) between the number of years between first and second vaccinations and the “take” rates. Sixty-two percent of respondents indicated they did not work with non–highly attenuated orthopoxviruses.

## Conclusions

In this study, laboratory workers continued to have successful “takes” (i.e., developed lesions) regardless of number of years since previous vaccination, suggesting that immunity might have waned. Therefore, our data do not provide evidence to suggest that the ACIP recommended interval for revaccination be prolonged. Although most respondents reported having no adverse effects from the vaccine, for some this vaccination caused discomfort. Many reported symptoms related to the occlusive dressing worn as a precautionary measure to ensure that the lesion site was properly covered during work hours. Other measures to ensure the lesion is covered appropriately, such as nonocclusive dressings and long sleeves, may be considered given that laboratory workers do not have direct contact with patients.

Although the LRN asks this small group of laboratory workers to comply with the ACIP recommendations, the question remains whether this requirement should include laboratory workers who do not handle orthopoxviruses. Revaccination of most laboratory workers at variola testing sites every 3 years would be expected to be sufficient to provide an initial immunologic response, whereas laboratory workers who do not handle orthopoxviruses could be vaccinated in the same fashion as other health care and public health workers who have at least 1 recent (since 2003) documented successful vaccination (*5*). This recommendation is based on the same premise as using the vaccine as prophylaxis for documented exposure to a smallpox-infected person. This practice was used regularly during the smallpox eradication program. Because the average incubation period for vaccinia is 3–4 days shorter than the incubation period for smallpox, a person exposed to smallpox would have a 3–4 day window in which to be vaccinated with and immunologically respond to vaccinia, which also confers immunity to smallpox (*6*)

Compromised immune systems or cardiac risk factors that make vaccinees ineligible for vaccination are more likely to develop as they age (*7*). Most barriers to revaccination were related to medical conditions (compromised immunity and/or exfoliative skin disorders) that place vaccinees at high risk for adverse events to the currently licensed smallpox vaccine. The conditions are an added challenge for the aging pool of laboratory workers assigned to national variola testing sites (*8*). Currently unlicensed third-generation smallpox vaccines may be considered (pending licensure) as replacements to ACAM2000 (Sanofi Pasteur Biologics, Lyon, France), the currently licensed vaccinia vaccine, for laboratory workers at national variola testing sites or perhaps an even broader population of laboratory workers throughout the United States. Third-generation vaccines are nonreplicating and safer in populations that might have contraindications to traditional vaccines (*9*–*11*).

The risk to the US population from a release of smallpox has decreased considerably. This reduced risk stems not from a lower threat from terrorism but from the existence of a stockpile of the new ACAM2000 smallpox vaccine, in addition to a cadre of health care and public health professionals who could be revaccinated quickly and mobilized accordingly (*12*).

More research on the immunogenicity of smallpox vaccine is needed but is challenged by the absence of smallpox disease to test the efficacy of vaccination. Researchers now appreciate that the complex mechanism of the immune response to vaccinia and/or smallpox infection might lead to better treatment options for infectious and autoimmune diseases (*7*). Future opportunities may arise to challenge the vaccine with the actual virus to measure vaccine efficacy and provide sound recommendations to protect all public health and health care responders against smallpox (*13*). In the meantime, ensuring that recommendations created to protect some populations are properly interpreted and applied is important to protecting the most vulnerable persons without exposing others to unnecessary harm.
